# On‐Surface Synthesis of Organolanthanide Sandwich Complexes

**DOI:** 10.1002/advs.202308125

**Published:** 2024-04-12

**Authors:** Shanmugasibi K. Mathialagan, Sofia O. Parreiras, Maria Tenorio, Lenka Černa, Daniel Moreno, Beatriz Muñiz‐Cano, Cristina Navío, Manuel Valvidares, Miguel A. Valbuena, José I. Urgel, Pierluigi Gargiani, Rodolfo Miranda, Julio Camarero, José I. Martínez, José M. Gallego, David Écija

**Affiliations:** ^1^ Instituto Madrileño de Estudios Avanzados en Nanociencia (IMDEA Nanoscience) Madrid 28049 Spain; ^2^ Brno University of Technology Brno 60190 Czech Republic; ^3^ ALBA Synchrotron Light Source Barcelona 08290 Spain; ^4^ Unidad de Nanomateriales Avanzados IMDEA Nanoscience Unidad Asociada al CSIC por el ICMM Madrid 28049 Spain; ^5^ Departamento de Física de la Materia Condensada and Condensed Matter Physics Center (IFIMAC) Universidad Autónoma de Madrid Cantoblanco Madrid 28049 Spain; ^6^ Instituto de Ciencia de Materiales de Madrid (ICMM) CSIC Cantoblanco Madrid 28049 Spain

**Keywords:** density functional theory, lanthanides, on‐surface synthesis, organometallic chemistry, STM/STS, XMCD

## Abstract

The synthesis of lanthanide‐based organometallic sandwich compounds is very appealing regarding their potential for single‐molecule magnetism. Here, it is exploited by on‐surface synthesis to design unprecedented lanthanide‐directed organometallic sandwich complexes on Au(111). The reported compounds consist of Dy or Er atoms sandwiched between partially deprotonated hexahydroxybenzene molecules, thus introducing a distinct family of homoleptic organometallic sandwiches based on six‐membered ring ligands. Their structural, electronic, and magnetic properties are investigated by scanning tunneling microscopy and spectroscopy, X‐ray absorption spectroscopy, X‐ray linear and circular magnetic dichroism, and X‐ray photoelectron spectroscopy, complemented by density functional theory‐based calculations. Both lanthanide complexes self‐assemble in close‐packed islands featuring a hexagonal lattice. It is unveiled that, despite exhibiting analogous self‐assembly, the erbium‐based species is magnetically isotropic, whereas the dysprosium‐based compound features an in‐plane magnetization.

## Graphical abstract

The growth of a new family of organometallic sandwich complexes prepared by on‐surface synthesis is demonstrated. These complexes are based on lanthanide atoms and partially deprotonated hexahydroxybenzene species. Their growth mechanism, structure, chemical configuration, electronic structure, and magnetic properties are disclosed.

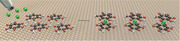

## 1 Introduction

Since the synthesis of the first organometallic single molecular magnet (SMM) in 2010,^[^ ^1^ ^]^ organolanthanide chemistry is an emerging field of research with great potential for the development of single molecular magnetism.^[^ ^2^ ^]^ However, the field is still in its infancy, and enormous research efforts are necessary to develop this new branch of chemistry, not only focusing on magnetic applications, but on its future potential in organic synthesis and catalysis.^[^ ^3^ ^]^

Theoretical calculations of organometallic compounds reveal promising magnetic properties. Metallocenes, for instance, exhibit remarkable magnetic anisotropy,^[^ ^4^ ^]^ while bimetallic benzene complexes are predicted to display unquenched orbital moments.^[^ ^5^ ^]^ In addition, 1D organometallic wires stand out for their predicted ferromagnetic or antiferromagnetic coupling.^[^ ^6^ ^]^

Experimentally, organometallic sandwich complexes are typically synthesized by solution chemistry methods, as is the case of metallocenes^[^ ^7^ ^]^ and other complex macromolecules.^[^ ^8^ ^]^ A few lanthanide‐based sandwich compounds prepared by this method were found to be good candidates for single molecular magnets. They can present slow relaxation times, magnetic remanence, high blocking temperature, and large anisotropy.^[^ ^9^ ^]^

Contemporarily, during the last two decades, on‐surface synthesis has emerged as a disruptive field of research that allows the preparation of low‐dimensional nanomaterials, whose potential applications include optoelectronics, spintronics, quantum information, sensing, and catalysis, among others.^[^ ^10^ ^]^ Many of these materials are typically precluded in wet chemistry and might include metal‐organic, organometallic, or covalent bonds.^[^ ^11^ ^]^

On‐surface synthesis has been extensively used for the fabrication of novel carbon‐based materials with 0D, 1D, or 2D dimensionality. Some examples are nanographenes,^[^ ^12^ ^]^ 1D polymers,^[^ ^13^ ^]^ and 2D covalent networks.^[^ ^14^ ^]^ In addition, it has revealed potential for the engineering of low‐dimensional metal‐organic nanoarchitectures, by linking organic ligands and metal nodes, in which molecular linkers could be coordinated with 3d^[^ ^15^ ^]^ or 4f^[^ ^16^ ^]^ metals.

One important advantage of this synthetic method is the design of defect‐free nanoarchitectures with long‐range ordering, as typically observed for 2D metal‐organic networks.^[^ ^17^ ^]^ However, it is important to take into account that, for both metal‐organic and organometallic materials, the metal atoms can interact with the substrate due to their close proximity to the surface. Such interaction can modify the electronic and magnetic properties of these materials, especially in the case of metallic substrates.^[^ ^18^ ^]^ For example, charge transfer and orbital hybridization can create interface states^[^ ^19^ ^]^ or reduce the magnetic moments.^[^ ^20^ ^]^ In this context, sandwich complexes are particularly interesting since their structure isolates the metallic atom, reducing its interaction with the surface while, at the same time, retaining the capability to form nanomaterials upon adsorption by self‐assembly.

While on‐surface chemistry has proven to be highly successful in designing 2D metal‐organic structures,^[^ ^17^, ^18^ ^]^ its application to organometallic sandwich architectures remains limited. A notable exception is the design of organometallic wires based on cyclooctatetraene (COT) ligands and Europium, featuring edge‐on adsorption of the molecular species and exhibiting ferromagnetism.^[^ ^21^ ^]^

**Scheme 1:**
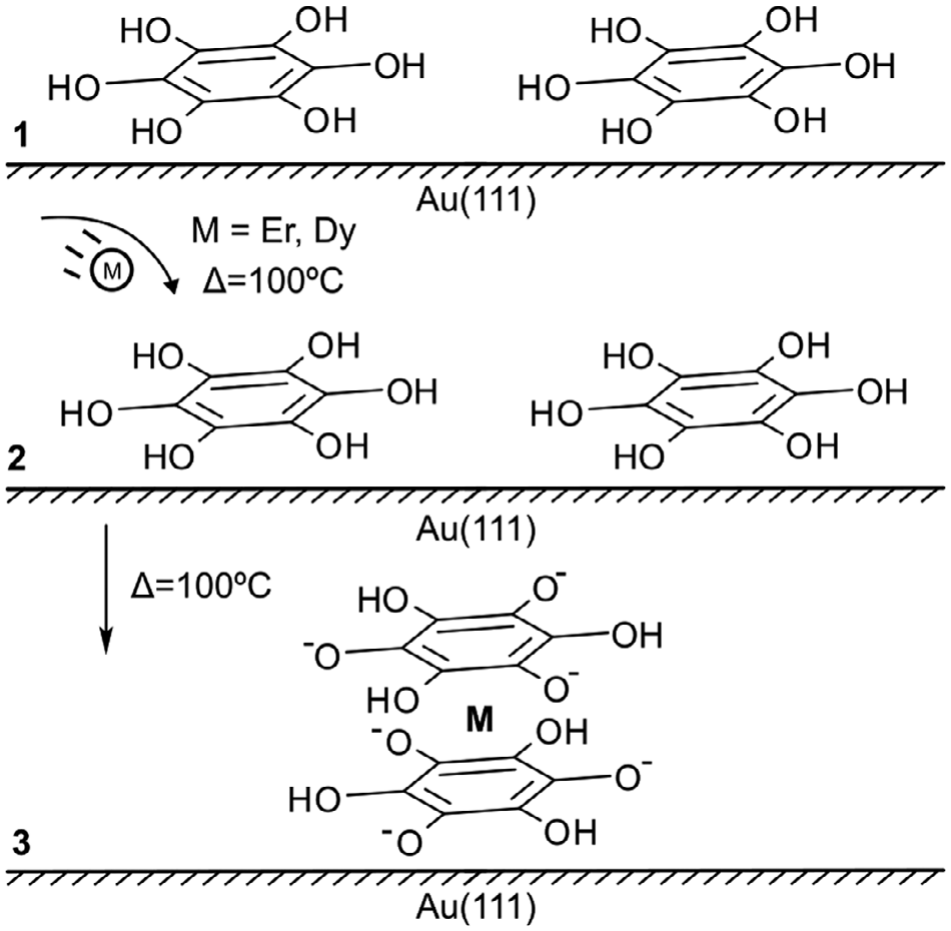
Chemical route to prepare Er(p‐HOB)_2_ or Dy(p‐HOB)_2_ species on Au(111). Some ligands in panel (**1**) might already be partially deprotonated at room temperature.^[^ ^22^ ^]^

Here, we take advantage of on‐surface synthesis protocols and the rich chemical properties of lanthanides (Er or Dy), reporting an unprecedented design of organometallic mononuclear sandwich complexes which are adsorbed flat on a Au(111) surface. Such compounds are based on lanthanide atoms (Er/Dy) sandwiched between partly deprotonated hexahydroxybenzene (H_6_HOB) ligands on Au(111), to be termed Er(p‐HOB)_2_ or Dy(p‐HOB)_2_, where p stands for partial deprotonation (see **Scheme** **1**).

H_6_HOB species (see panel **1** of Scheme 1) have been previously used in the preparation of surface‐confined metal‐organic nanoarchitectures by on‐surface synthesis with transition metal atoms.^[^ ^22^, ^23^ ^]^ The crucial difference in our study resides in the use of lanthanides (Er and Dy), which are able to form sandwich complexes with H_6_HOB species on Au(111). Herein, the confined growth on the surface leads to the self‐assembly of a periodic hexagonal network of novel organometallic sandwiches. Thus, here we report, to the best of our knowledge, the first use of lanthanides to engineer organometallic sandwich species on surfaces.

The structural, electronic, and magnetic properties of the organolanthanide complexes were investigated in a multidisciplinary study that combined scanning tunneling microscopy (STM), scanning tunneling spectroscopy (STS), X‐ray absorption spectroscopy (XAS), X‐ray magnetic circular dichroism (XMCD), X‐ray linear dichroism (XLD), and X‐ray photoelectron spectroscopy (XPS). The experimental results were complemented by density functional theory (DFT) calculations and STM‐imaging simulations. Our results demonstrate that it is feasible to prepare periodic nanoarchitecture of mononuclear organolanthanide sandwich complexes on Au(111). Importantly, the exchange of the lanthanide metal (Er vs Dy) preserves the architecture but results in distinct magnetic properties.

## 2 Results and Discussion

### 2.1 On‐Surface Synthesis and Structural Characterization

**Figure 1:**
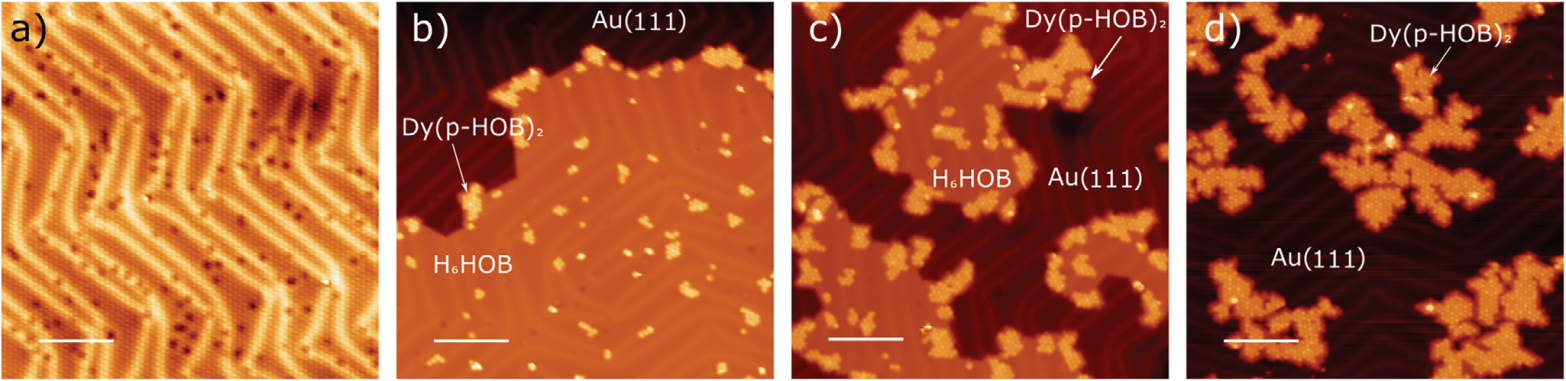
Sequential addition of dysprosium atoms on H_6_HOB/Au(111). a) STM image of only H_6_HOB molecules deposited on a clean Au(111) substrate held at room temperature. From b) to d) STM images of sequential sublimation of Dy on a Au(111) substrate with close to a monolayer of H_6_HOB molecules. Total Dy sublimation time: 1 min (b), 3 min (c), and 5 min (d). In all preparations, the substrate was held at 100 °C during Dy deposition, and the samples where post‐annealed at 100 °C for 10 min. The scale bar is 10 nm in (a) and 12 nm in (b–d). Scanning parameters: a) V_bias_ = 0.5 V, I_t_ = 20 pA, T = 4 K; b) V_bias_ = 0.5 V, I_t_ = 50 pA, T = 4 K; c) V_bias_ = 0.5 V, I_t_ = 10 pA, T = 4 K; d) V_bias_ = 1.0 V, I_t_ = 20 pA, T = 4 K.

The organolanthanide sandwich complexes were synthesized following a three‐step procedure (see scheme 1). In the first step, the H_6_HOB molecules are sublimated on a clean Au(111) surface at room temperature (RT), with a coverage close to one monolayer, as shown in **Figure** **1**a. The molecular species, visualized as circular protrusions, form a close‐packed hexagonal self‐assembly. Afterward, lanthanide atoms (Dy or Er) are deposited on the sample held at 100 °C. Finally, the sample is annealed for 10 min at the same temperature (100 °C). This procedure gives rise to a nanoarchitecture containing organolanthanide sandwich complexes, displayed in panel 3 of Scheme 1, in which, according to XPS measurements, the hexahydroxybenzene rings are partially deprotonated (see Figure S1, Supporting Information, and related discussion).

**Figure 2:**
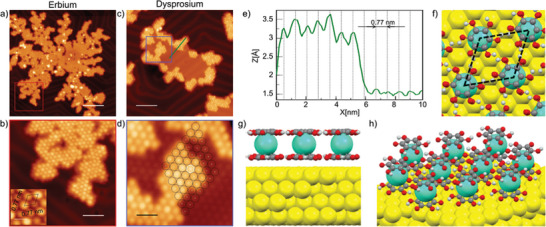
Lanthanide‐based sandwich complexes on Au(111). a) STM image overview of as‐grown Er(p‐HOB)_2_, with a close view of the island framed in a red square and displayed in b). The inset of (b) is a zoom‐in of the sandwich structure with the unit cell represented by a rhombus. The bar scale is 1 nm. c) STM image overview of partially grown Dy(p‐HOB)_2_. d) A closer view of the purple frame is displayed in c) with a superimposed model (black circles) centered on a second layer molecule (white circle) showing that the second layer molecules are perfectly aligned with the first layer molecules. e) Height profile of the green line in d), in which the X = 0 point from the profile corresponds to the upper right end of the line. f–h) vdW‐DFT‐D3 calculation of the preferred adsorption geometry of the Dy(p‐HOB)_2_. (f) top view showing the unit cell in dashed lines; g,h) the side views in (g) showing the partial deprotonation of the H_6_HOB molecules. Yellow, cyan, red, grey, and white balls represent Au, Dy, O, C, and H atoms, respectively. Scale bars are: a) 14 nm, b) 4 nm, c) 8 nm, d) 2 nm. Scanning parameters: a) V_bias_ = 0.5 V, I_t_ = 10 pA, T = 4 K; b) V_bias_ = 0.5 V, I_t_ = 10 pA, T = 4 K, c) V_bias_ = 0.4 V, I_t_ = 10 pA, T = 4 K; d) V_bias_ = 0.4 V, I_t_ = 10 pA, T = 4 K.

Figure 1b displays an STM image of a sample prepared following the above‐mentioned protocol after the deposition of 1 min of Dy and subsequent post‐annealing. Remarkably, it can be observed that some H_6_HOB precursors are placed on top of the molecular submonolayer, forming a second layer. In addition, the spatial extension of the first monolayer has decreased as compared to the initial stage before depositing lanthanides (see Figure 1a). When an additional amount of Dy (2 more minutes at the same flux) is further deposited and the sample is post‐annealed at 100 °C for 10 min, more molecules are displaced to the second layer (see Figure 1c). If this procedure is repeated, with the deposition of 2 more minutes of Dy, the second layer increases notably, signaling the almost disappearance of the first layer. If the same procedure is followed, but exchanging the metal atom for Er, a similar structure is formed (see **Figure** **2**a,b). A tentative explanation for such phenomenon could be the formation of organolanthanide sandwiches, as we will corroborate in the following observations.

First, it is important to point out that when annealing at a temperature of 100 °C for 10 min, the molecular monolayer is not desorbed. However, by annealing at slightly higher temperature, e.g. 125 °C, the first layer desorbs, while the double layer remains intact (see Figure S2, Supporting Information). The desorption of the double layer only occurs when annealing at much higher temperatures, above 200 °C. Second, a careful analysis of the STM images indicates that the second molecular layer is aligned with the first layer (see Figure 2d and line profile in Figure 2e). The STM apparent heights are ≈ 1.65 Å for the first layer and ≈ 3.32 Å for the second layer, i.e. the second layer is apparently twice as high as the first one at 0.4 V. However, depending on the scanning parameters it can be observed that some molecules are slightly brighter than others (see Figure S5c, Supporting Information).

As a proof‐of‐concept, we have performed a comprehensive series of thermochemical atomistic simulations, based on DFT combined with the Gibbs free‐energy formalism, involving the calculation of minimum reaction paths, transition states, and energy barriers, about the on‐surface formation of the partially deprotonated Dy(p‐HOB)_2_ and the pristine Dy(HOB)_2_ species. The results of these calculations establish viable mechanisms toward the on‐surface formation of both pristine and partially deprotonated species, predicting the preferential on‐surface formation of the Dy(p‐HOB)_2_ species within the temperature range of this study, in excellent agreement with the experimental evidence (see further details in Figure S3, Supporting Information). Figure 2f,g shows DFT‐optimized atomic models for Dy(p‐HOB)_2_ on Au(111). As will be discussed later, the STM images seem to indicate that the molecules present aleatory degrees of deprotonation. However, in order to facilitate the calculations, it was considered that all molecules lost half of their H (as observed on average by XPS, Figure S1, Supporting Information).

The results of the calculations are in agreement with partial deprotonation of the molecules (see Figures S4 and S5, Supporting Information), with the molecules physisorbed, lying at 3.35 Å above the surface. The adsorption energy is 0.12 eV per molecule and, after testing all the inequivalent adsorption sites, the preferential adsorption site is with the center of the C ring on‐hollow position. Moreover, a comparison of simulated and experimental STM images indicates that a possible explanation for the molecules with different brightness observed in the STM images is the coexistence of organometallic sandwiches with distinct degrees of deprotonation^[^ ^24^ ^]^ (see Figure S5, Supporting Information, and related discussion).

Disregarding the different brightness of the molecules, the unit cell is represented by dashed lines in the inset of Figure 2b: the intermolecular distance is 0.77 nm and the lattice vectors are related to the lattice vectors of the Au surface by the epitaxial relationship $\left(\begin{array}{cc} 3 & 1\\ -1 & 2 \end{array}\right)$. These results are in excellent agreement with the experiments, where the intermolecular distance is (0.73 ±  0.04) nm.

Additionally, DFT calculations were performed for a Dy atom positioned underneath the molecule, i.e. between the molecule and the substrate (Figure S6, Supporting Information). In this case, the system is not stable for any initial configuration.

Altogether, the experimental and theoretical results indicate that the on‐surface synthesis procedure described above leads first to the formation of organolanthanide sandwich complexes, in which Dy/Er atoms are sandwiched between two partially deprotonated species. Next, Er(p‐HOB)_2_ or Dy(p‐HOB)_2_ compounds self‐assemble in molecular islands featuring a hexagonal lattice, which are stabilized by supramolecular interactions, predominantly hydrogen bonds between adjacent organolanthanide sandwiches. Interestingly, the preparation of sandwich complexes on surfaces based on porphyrin or phthalocyanine backbones was reported previously. However, such systems are metal‐organic complexes (i.e., there is no metal‐C bond).^[^ ^25^ ^]^ To the best of our knowledge, the on‐surface synthesis of organometallic sandwich complexes is unprecedented.

### 2.2 Electronic Properties

**Figure 3:**
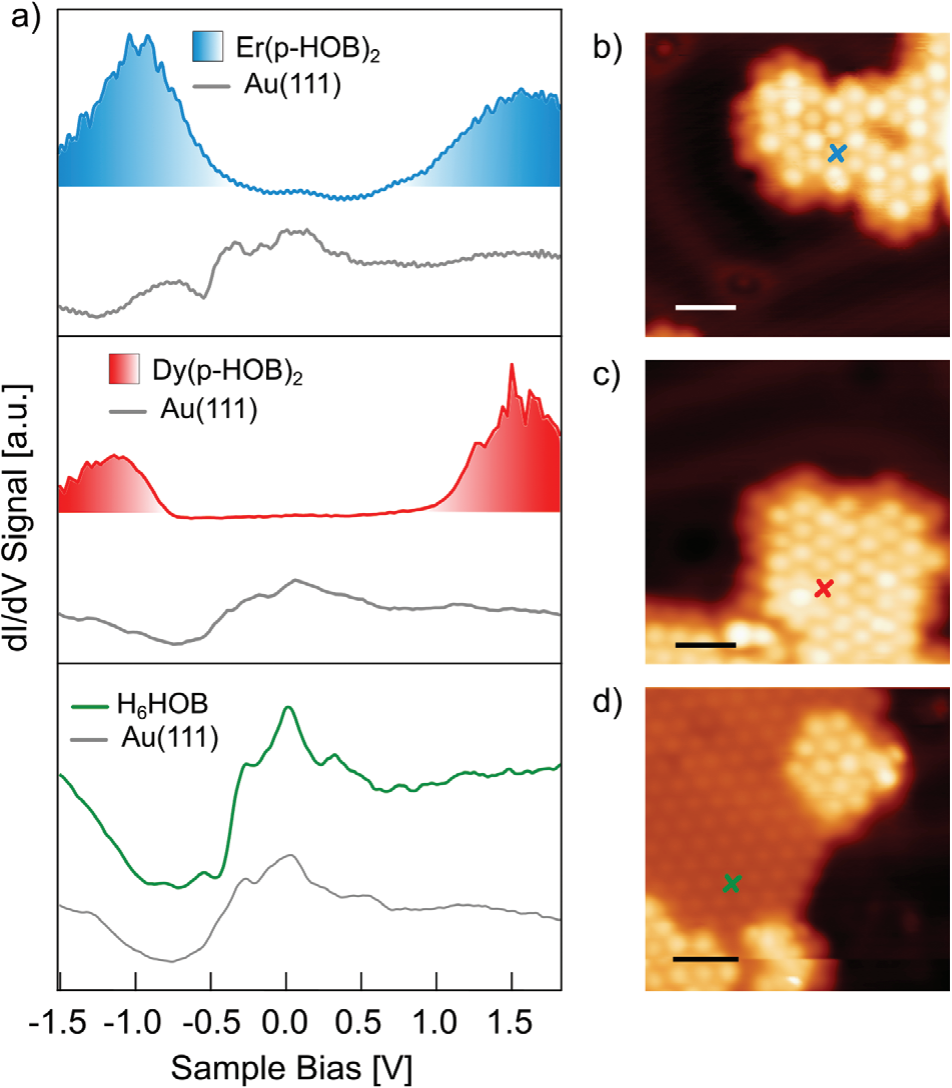
Characterization of the electronic properties of Er(p‐HOB)_2_ and Dy(p‐HOB)_2_. a) From top to bottom, STS of Er(p‐HOB)_2_ (blue plot), Dy(p‐HOB)_2_ (red plot), and H_6_HOB molecules (green plot) on Au(111). The Au(111) reference taken at a closer point is presented below every spectrum (grey plots). b–d) STM images of the location of the taken STS spectra. Scale bars are 2 nm in all STM images. Scanning parameters: b) V_bias_ = 500 mV, I_t_ = 30 pA, T = 4 K; c) V_bias_ = 500 mV, I_t_ = 50 pA, T = 4 K; d) V_bias_ = 500 mV, I_t_ = 40 pA, T = 4 K.

Characterization of the electronic properties of the organolanthanide complexes was performed by STS measurements. The main results are presented in **Figure** **3**. For both Er(p‐HOB)_2_ and Dy(p‐HOB)_2_ compounds two prominent peaks are observed at negative and positive bias, which are assigned to the highest occupied molecular orbital (HOMO) and the lowest unoccupied molecular orbital (LUMO), respectively. The spikes observed in the resonances of both Er(p‐HOB)_2_ and Dy(p‐HOB)_2_ are attributed to inelastic excitations of the molecular species, which could be related to a rotation or bending of the top decker with respect to the bottom one. It was also observed that the positions of the HOMO and LUMO resonances can shift when measured under the same conditions (see Figures S7–S9, Supporting Information). The positions for the HOMO peaks range from –0.3 to –1.7 eV for Er(p‐HOB)_2_ and from –0.7 to –1.5 for Dy(p‐HOB)_2_; whereas the LUMO peaks range from 0.6 to 1.6 eV for Er(p‐HOB)_2_ and from 0.9 to 1.8 eV for Dy(p‐HOB)_2_. The electronic bandgaps, defined from the onsets of the resonances, range from 0.5 to 1.5 eV for Er(p‐HOB)_2_ and 1.0 to 1.7 eV for Dy(p‐HOB)_2_. For the p‐HOB single layer, on the other side, the STS spectra are very similar to the spectra of the Au(111) surface, and no extra resonance could be detected between –2 and +2 eV. The shifts in the resonance positions and bandgaps could be attributed to the distinct degrees of deprotonation, registry, and conformational degrees of freedom.

### 2.3 Magnetic Properties

**Figure 4:**
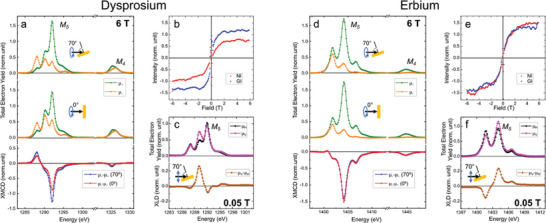
Magnetic characterization of Dy(p‐HOB)_2_ and Er(p‐HOB)_2_ species on Au(111). a–c) Dy(p‐HOB)_2_; d–f) Er(p‐HOB)_2_. a,d) XAS with positive (µ_+_, green) and negative (µ_‐_, orange) circularly polarized light and XMCD (µ_‐_ − µ_+_) taken at the Dy/Er M_4,5_‐edges at normal (0°, red) and grazing (70°, blue) incidences (B = 6 T, T = 1.6 K). b,e) Magnetization curves constructed by measuring the XMCD intensity at the most intense peak of the Dy/Er M_5_‐edge. (T = 1.6 K). c,f) XAS spectra acquired with vertical (µ_V_, pink) and horizontal (µ_H_, black) linearly polarized light and XLD (µ_V_ – µ_H_, brown) taken at the Dy/Er M_5_‐edge at grazing (70°) incidence (B = 0.05 T, T = 1.6 K).

The magnetic characterization has been performed in the BOREAS beamline at the ALBA synchrotron^[^ ^26^ ^]^ by means of XAS, XLD, and XMCD experiments. **Figure** **4**a,d present XAS and XMCD spectra measured at 6 T for normal (NI, 0°) and grazing (GI, 70° with respect to the surface normal) incidences for Dy(p‐HOB)_2_ and Er(p‐HOB)_2_. The peak structures of both systems are characteristic of a +3 oxidation state,^[^ ^27^ ^]^ which is in agreement with the results of the DFT calculations, where the Dy atoms hold a charge of +2.83 |e‐|.

For Dy(p‐HOB)_2_ complexes, the XMCD has a higher intensity at GI (see Figure 4a), which is indicative of an in‐plane magnetization easy axis and further confirmed by the fact that the magnetization curves reach saturation at a lower magnetic field at GI than at NI (see Figure 4b; Figure S10, Supporting Information). In the case of the Er(p‐HOB)_2_, the XMCD intensity and the magnetization curves are very similar at GI and NI (Figure 4d,e). This can be indicative of a negligible magnetic anisotropy or a tilted magnetization easy axis.^[^ ^28^ ^]^

The XLD spectrum of Dy(p‐HOB)_2_ measured at GI (Figure 4c) is very similar, both in shape and intensity, to the XLD of Dy‐TDA metal‐organic networks,^[^ ^29^ ^]^ where a close to in‐plane anisotropy was observed. For Er(p‐HOB)_2_, the XLD spectrum (Figure 4f) has lower intensity than for Er‐TDA networks,^[^ ^30^ ^]^ where an out‐of‐plane anisotropy was observed; and resembles more the spectrum of Er‐DBPB networks^[^ ^28^ ^]^ that present a tilted magnetization easy axis.

We can compare these results with other sandwich‐type complexes previously reported. Dy double‐deckers (DyPc_2_) have an out‐of‐plane anisotropy,^[^ ^10c^ ^]^ while for ErPc_2_ the anisotropy is in‐plane.^[^ ^31^ ^]^ The differences between our results and the previous reports for double‐deckers can be explained by the differences in the crystal field geometry. We have previously demonstrated that small variations in the crystal field can lead to significant changes in the magnetic properties.^[^ ^29^ ^]^ In the case of double‐deckers, the geometry of the ligands forms a square antiprism where the lateral distance and the height are similar,^[^ ^32^ ^]^ and effectively acts as a coordination sandwich as described by Rinehart and Long,^[^ ^33^ ^]^ compressing the lanthanide atom, which would favor a contraction of the charge toward the plane (see Figure S11, Supporting Information). This favors an out‐of‐plane alignment of spins for Dy^3+^ (oblate ions) and an in‐plane alignment for Er^3+^ (prolate ions). However, for Er(p‐HOB)_2_ and Dy(p‐HOB)_2_ reported here, the lateral size of the six‐member ring is 2.8 Å while the vertical distance between the two tapes is 4.1 Å, around four times larger than the diameter of the ionic radii of Ln^3+^ ions (≈1.1 Å). So, in this case, the effective crystal field is a very elongated antiprism that actually has the opposite effect of favoring an out‐of‐plane orientation of the charge (see Figure S11, Supporting Information). This effect can be observed in the opposite orientation of the XLD spectra of Dy(p‐HOB)_2_ when compared to the double‐deckers^[^ ^6c^ ^]^ and leads to the in‐plane magnetic anisotropy observed for the oblate ions in this crystal field. Similarly, for the Er prolate ions the crystal field favors an out‐of‐plane magnetic anisotropy.

The expectation values of the magnetic moments at the experimental conditions of 1.6 K and 6 T, presented in **Table** **1**, were calculated by sum‐rule analysis of the XMCD results. For Er(p‐HOB)_2_ there is a small difference in the magnetic moments measured at NI and GI incidence, with slightly higher values at NI and a *J_z_* of 11/2. Dy(p‐HOB)_2_ has a larger anisotropy, presenting higher magnetic moments at GI and the *J_z_* value is also 11/2.

**Table 1:** Expectation values of the effective spin (〈*S_z_* 〉), orbital (〈*L_z_* 〉) and total (〈*J_z_* 〉 = 〈*S_z_* 〉  + 〈*L_z_* 〉) moments, and total magnetic moment (*m_T_* =  2〈*S_z_* 〉 + 〈*L_z_* 〉), calculated by XMCD sum‐rule analysis, for grazing (70°) and normal (0°) incidences at 1.6 K and 6 T for Dy(p‐HOB)_2_ and Er(p‐HOB)_2_ on Au(111).

	Dy(p‐HOB)_2_		Er(p‐HOB)_2_	
Incidence angle (°)	70	0	70	0
〈*S_z_* 〉	1.8(2)	1.1(1)	0.9(1)	1.0(1)
〈*L_z_* 〉	3.7(4)	2.3(2)	3.6(4)	4.2(4)
〈*J_z_* 〉	5.5(6)	3.4(3)	4.5(5)	5.3(5)
*m_T_*	7.3(8)	4.6(4)	5.3(6)	6.3(6)

## 3 Conclusion

In summary, we report the on‐surface synthesis of partially deprotonated organolanthanide sandwich complexes on an Au(111) surface, i.e. Dy(p‐HOB)_2_ and Er(p‐HOB)_2_ species. The sequential deposition of H_6_HOB linkers at room temperature, and Dy or Er atoms with the substrate held at 100 °C, followed by an annealing, leads to the formation of sandwich‐type complexes, in which each lanthanide atom is positioned in between two partially deprotonated linkers. Such compounds self‐assemble into close‐packed supramolecular layers featuring a hexagonal lattice. Despite the similar electronic structure of the Dy(p‐HOB)_2_ and Er(p‐HOB)_2_ complexes, both compounds display different magnetic properties, which can be rationalized by an effective very elongated antiprism crystal field. For Dy(p‐HOB)_2_ the magnetization easy‐axis lies in the surface plane, while for Er(p‐HOB)_2_ an almost isotropic magnetization was observed.

Our results open avenues for the development of organometallic chemistry on surfaces. Furthermore, our findings envision the capability to tailor the magnetic properties of such complexes on surfaces, while preserving the structural integrity by lanthanide exchange.

## Conflict of Interest

The authors declare no conflict of interest.

## Supporting information

Supporting Information

## Data Availability

The data that support the findings of this study are openly available in https://repositorio.imdeananociencia.org.
