# Supplementary material for: On‐Surface Synthesis of Organolanthanide Sandwich Complexes
Source: Adv Sci (Weinh). 2024 Apr 12;11(24):2308125. doi: 10.1002/advs.202308125 (PMC11200025; doi:10.1002/advs.202308125)
Supplement: Supplementary file 1 — Supporting Information [file ADVS-11-2308125-s001.pdf]

ADVANCED SCIENCE
Open Access

Supporting Information

for *Adv. Sci.*, DOI 10.1002/advs.202308125

On-Surface Synthesis of Organolanthanide Sandwich Complexes

Shanmugasibi K. Mathialagan, Sofia O. Parreiras, Maria Tenorio, Lenka Černa, Daniel Moreno, Beatriz Muñiz-Cano, Cristina Navío, Manuel Valvidares, Miguel A. Valbuena, José I. Urgel, Pierluigi Gargiani, Rodolfo Miranda, Julio Camarero, José I. Martínez, José M. Gallego and David Écija**

Supporting Information
©Wiley-VCH 2021
69451 Weinheim, Germany

On-surface synthesis of organolanthanide sandwich complexes

Shanmugasibi K. Mathialagan,† Sofia O. Parreiras,*† Maria Tenorio, Lenka Černa, Daniel Moreno, Beatriz Muñiz-Cano, Cristina Navío, Manuel Valvidares, Miguel A. Valbuena, José I. Urgel, Pierluigi Gargiani, Rodolfo Miranda, Julio Camarero, José I. Martínez, José M. Gallego, and David Écija*

Abstract: The synthesis of lanthanide-based organometallic sandwich compounds is very appealing regarding their potential for single molecule magnetism. Here, it is exploited by on-surface synthesis to design unprecedented lanthanide-directed organometallic sandwich complexes on Au(111). The reported compounds consist of Dy or Er atoms sandwiched between partially deprotonated hexahydroxybenzene molecules, thus introducing a distinct family of homoleptic organometallic sandwiches based on six-membered ring ligands. Their structural, electronic, and magnetic properties are investigated by scanning tunneling microscopy and spectroscopy, X-ray absorption spectroscopy, X-ray linear and circular magnetic dichroism, and X-ray photoelectron spectroscopy, complemented by density functional theory-based calculations. Both lanthanide complexes self-assemble in close-packed islands featuring a hexagonal lattice. It is unveiled that, despite exhibiting analogous self-assembly, the erbium-based species is magnetically isotropic, whereas the dysprosium-based compound features an in-plane magnetization.

DOI: 10.1002/advs.202308125

Table of Contents

Table of Contents 1
Experimental Procedures 2
Theoretical Framework 3
Results and Discussion 4
References 14
Author Contributions 14

Experimental Procedures

The scanning tunnelling microscopy (STM) experiments were carried out at Fundación IMDEA Nanociencia, in an ultra-high vacuum setup with a base pressure of $5x10^{-10}$ mbar. The microscope is a low-temperature Scienta-Omicron Polar STM that works at 4.4 K. The Au(111) crystal used to prepare the samples was cleaned by repeated cycles of sputtering (Ar+, 1.5 KeV) and annealing (500 ºC).

The hexahydroxybenzene (H_6HOB) molecular precursors have been bought from *Tokyo Chemical Industry Co., LTD* (>98% of purity). They are sublimated in UHV via organic molecular beam epitaxy (OMBE) from a commercial Kentax Knudsen cell at 150 ºC, at a deposition rate of 0.33 ML/min on an already clean Au(111) crystal held at room temperature. The lanthanide atoms are sublimated from the respective metal rods from a commercial Focus (EFM3Ts) e-beam evaporator, with the substrate held at 100 ºC. As discussed in the main text, the samples were post-annealed at temperatures of 100 ºC or 125 ºC to form the sandwich structure.

Both STM topography and scanning tunneling spectroscopy (STS) data were acquired in the same Scienta-Omicron Polar STM at a base pressure of $1x10^{-10}$ mbar. The STS measurements were carried out at constant height, with a lock-in frequency of 933 Hz, a time constant of 13 ms, and a voltage modulation of 30 mV. The acquired data was post-processed in IgorPro program by applying a binary smoothing of order 3.

The C 1s and O 1s X-ray photoelectron spectroscopy (XPS) core level spectra were obtained from a Dy(p-HOB)$_2$/Au(111) sample, which was post-annealed at 125 ºC to ensure that all the non-coordinated molecules desorbed and the signal arises only from the sandwich structure. The spectra were taken at -130 ºC from a UHV system at IMDEA Nanociencia, holding a SPHERA-U7 hemispherical energy analyzer that uses a monochromatic X-ray source (Al K_α line with energy of 1486.71 eV). The samples were prepared in a separated STM chamber in order to check the surface coverage (0.3 ML) and transferred with a vacuum suitcase held at a pressure < 10^{-8} mbar to the XPS chamber (base pressure $4x10^{-10}$). After the XPS measurements, the sample was transferred back to the STM chamber to check for any possible damage. The spectra were fitted using XPST macro for IGOR (Dr. Martin Schmid, Philipps University Madburg). The Au $4f_{7/2}$ core level centered at 84.0 eV was taken as a binding energy reference. The line shape of the spectra was adjusted with Voigt functions [1] (Lorentzian-Gaussian profile, with a ratio of 0.3). Shirley and Tougaard backgrounds were used for C 1s and for O 1s peaks, respectively. No constraints were imposed on the fitting, and the best fit considered was the one with minimum R-factor. The atomic concentration calculation was obtained with CasaXPS software, and the results are displayed in Table S1.

The samples measured in the ALBA synchrotron light source (Cerdanyola del Vallès, Barcelona) were previously grown in the STM at IMDEA Nanociencia, and then carried to the synchrotron in an UHV suitcase at a base pressure of < $1x10^{-9}$ mbar. The polarization-dependent X-ray absorption spectroscopy (XAS), X-ray magnetic circular dichroism (XMCD) and X-ray linear dichroism (XLD) experiments performed at the synchrotron were done in the total electron yield (TEY) detection, with a 90% circularly polarized beam and applying magnetic fields up to 6 T in the direction of incidence of the X-ray beam. The signal was normalized to the incident X-ray flux measured as the TEY signal of a freshly-evaporated gold mesh placed between the last optical element and the sample. The beam spot size was defocused to about 4x8 HxV mm^2 FWHM in order to reduce the photon density on the sample and consequently lowering the beam damage. All experiments were carried out at a temperature of 1.6 K measured at the cold finger. The data was acquired by varying the photon energy at the $M_{4,5}$ edges of Erbium and Dysprosium, and the sample was rotated with respect to the normal direction of the X-ray beam 0º (normal incidence, NI) and 70º (grazing incidence, GI). XLD is defined as the difference between XAS spectra measured with vertical and horizontal polarizations ($\mu_V - \mu_H$). The spectra were measured at 0.05 T and 6 T and normalized to the maximum of Erbium and Dysprosium M_5-edge of the isotropic spectra: $XAS_{iso} = (\mu_V/3 + 2\mu_H/3)$. On the other hand, XMCD is obtained as the difference between the right-circular and the left-circular polarized light and normalized to the maximum of the Erbium and Dysprosium M_5-edge of the average absorption spectra: $XAS_{ave} = (\mu_+ + \mu_-)/2$. The magnetization curves are obtained by calculating at every magnetic field value, the difference between the maximum signal of the M_5-edge and its baseline (M_5 pre-edge) at a rate of 1 T/min. A Python script is run so that it automatically changes this magnetic field up to 6 T, and also allows to obtain the dichroic magnetic signal at each step.

Sum-rule analysis [2] was used to obtain expectation values of the quantum numbers $\langle L_z \rangle$ and $\langle S_z \rangle$ in units of $\hbar$, the total moment $\langle J_z \rangle = \langle L_z \rangle + \langle S_z \rangle$ in units of $\hbar$ and total magnetic moment $m_T = \langle L_z \rangle + 2\langle S_z \rangle$ in μ_B. The analysis considered a number of holes of 5 for Dy^{+3} and 3 for Er^{+3}. It was assumed a constant $\langle T_z \rangle / \langle S_z \rangle$ ratio of -0.053 for Dy^{+3} and +0.213 for Er^{+3} [3].

Theoretical Framework

All the ab initio calculations of the different pristine and deprotonated Dy@H_6HOB/Au(111) systems were carried out by using an adequate combination of the plane-wave QUANTUM ESPRESSO simulation package [4] to obtain optimized interfacial structures and electronic properties, with the efficient localized-basis set FIREBALL code [5] for the STM-imaging simulation.

Within the QUANTUM ESPRESSO code, one-electron wave functions were expanded using a plane-wave basis set, with energy cutoffs of 550 eV and 650 eV for kinetic energy and electronic density, respectively. To account for electronic exchange and correlation effects, the PBESol approximation, a revised generalized gradient-corrected functional, was employed [6]. This functional is known for its high accuracy in determining geometries, with interatomic distance errors below 0.5% compared to experimental values, and enhanced values of vibrational frequencies due to its precise representation of the gradient expansion for solids [7]. To accurately model the ion-electron interaction of the constituent atoms (H, C, O, Dy and Au), fully relativistic Kresse-Joubert projector-augmented wave pseudopotentials were used [8]. These pseudopotentials allow for the inclusion of spin-orbit coupling effects, particularly for Dy atoms, which were treated with 20 valence electrons ($5s^2 5p^6 4f^{10} 6s^2$) to account for the hypothetical role of f electrons in the interfacial chemistry. Long-range dispersion interactions were taken into account by applying a semi-empirical R^{-6} correction by the DFT+D3 implementation [9]. The Brillouin zone was sampled using optimal Monkhorst-Pack k-point grids [10]. All the calculations were performed by simultaneous cell + structure relaxation, with the Au(111) substrate modeled as an infinite 2D periodic slab consisting of four physical layers. The two bottommost layers were kept fixed during the geometrical optimizations. To prevent interaction between adjacent slabs, a 25 Å-thick vacuum region separated the systems in neighboring cells along the perpendicular-to-the-surface direction.

To compute the STM-imaging simulations, we used the local-orbital formulation of DFT as implemented in FIREBALL (ref. [5] and references therein). This approach incorporates self-consistency on the orbital occupation numbers [11], which are obtained using orthonormal Löwdin orbitals. Tunneling currents for the STM images were computed using the Keldysh-Green function formalism for the obtained optimal geometries of interfacial systems studied. The first-principles tight-binding Hamiltonian, obtained from the FIREBALL code, was employed for these computations (detailed explanations can be found elsewhere [12]), and, importantly, both sample and tip contributions were explicitly treated. All theoretical STM images were simulated under constant-current scanning conditions to replicate the experimental procedure. The tunneling current and bias voltage were set at I_t = 0.1 nA and V_{bias} = +0.5 V, respectively.

Results and Discussion

I. XPS data and discussion

The O 1s core level peak showed two components, one at 533 eV coming from the –OH group, and another one at 531 eV that is associated to the $–CO^-$ group [13]. We attribute these two peaks to a partial deprotonation of the H_6HOB molecules after the mild annealing to 125 ºC, as reported in previous works for molecules with hydroxyl functional groups [13]. The integrated area of the two O components is approximately the same, indicating that ~50 % of the O atoms are deprotonated. In the C 1s XPs core level peak, this distinction cannot be done due to the limited resolution, since the two peaks implied (C-OH and CO) should be separated 0.2 eV in energy [13a].

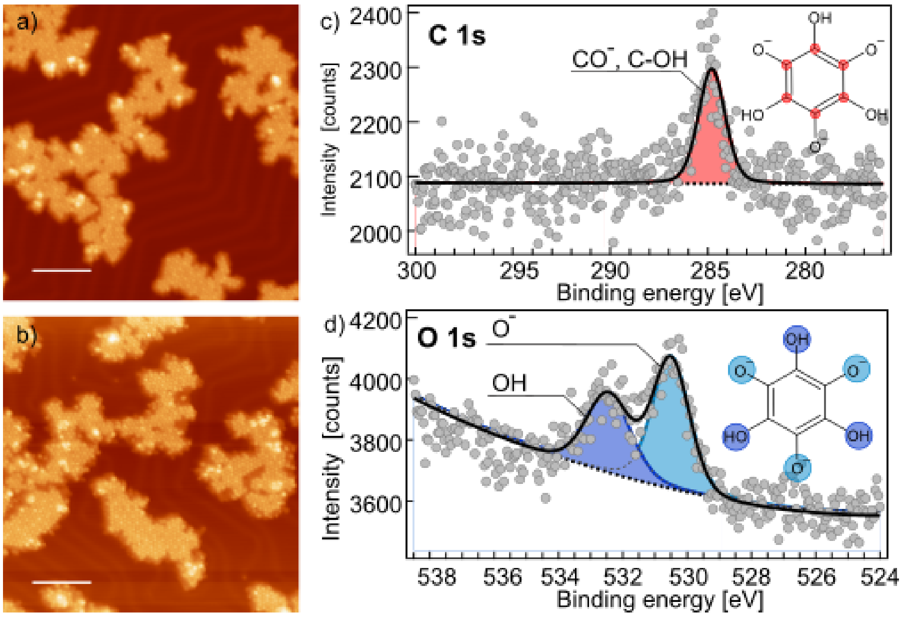

Figure S1: Dy(p-HOB)$_2$ STM and XPS analysis. (a) STM image of Dy(p-HOB)$_2$ before transferring it to the XPS chamber. (b) Same sample after being transferred from the XPS to the STM chamber in order to check its stability. (c-d) C 1s and O 1s core level XPS spectra from the Dy(p-HOB)$_2$ sample. C 1s main peak is present at 284.9 eV (c), whereas O 1s shows contribution from OH at 532.5 eV and from O^- at 530.5 eV (d). Atomic concentration calculation of each species is displayed in Table S5. a-b) Scale bar = 10 nm. Scanning parameters of the STM images: (a): V_{bias} = 0.3 V, I_t = 50 pA, T = 4 K; (b): V_{bias} = 0.5 V, I_t = 50 pA, T = 4 K.

Table S1. Calculation of atomic concentrations from C 1s and O 1s core level XPS spectra.

Element	BE average [eV]	Relative area	FWHM [eV]
C 1s	285.4	52.8%	1.51
O 1s (OH)	533.0	24.0%	2.10
O 1s (O-)	531.0	23.2%	1.37

II. Stability of the supramolecular architectures as a function of the temperature.

As described in the main text, in order to remove the non-coordinated molecules, a further post-annealing to 125 ºC is carried out. To demonstrate so, we performed two different control experiments that are depicted in Figure S2. In one of the experiments, H_6HOB molecules and lanthanide atoms are added in the usual way to grow the organometallic sandwich complexes (Figure S2a). This system is further annealed in a stepwise manner from RT to 225 ºC and checked in the STM. Another control experiment is performed by depositing only H_6HOB molecules, with no lanthanides (Figure S2b), and then annealed at mild temperatures (125º C). As it can be seen only the sample prepared with lanthanide atoms shows no desorption of the species up to annealing temperatures of 225 ºC. On the other hand, in the absence of lanthanide atoms, molecular desorption already takes place at 125 ºC.

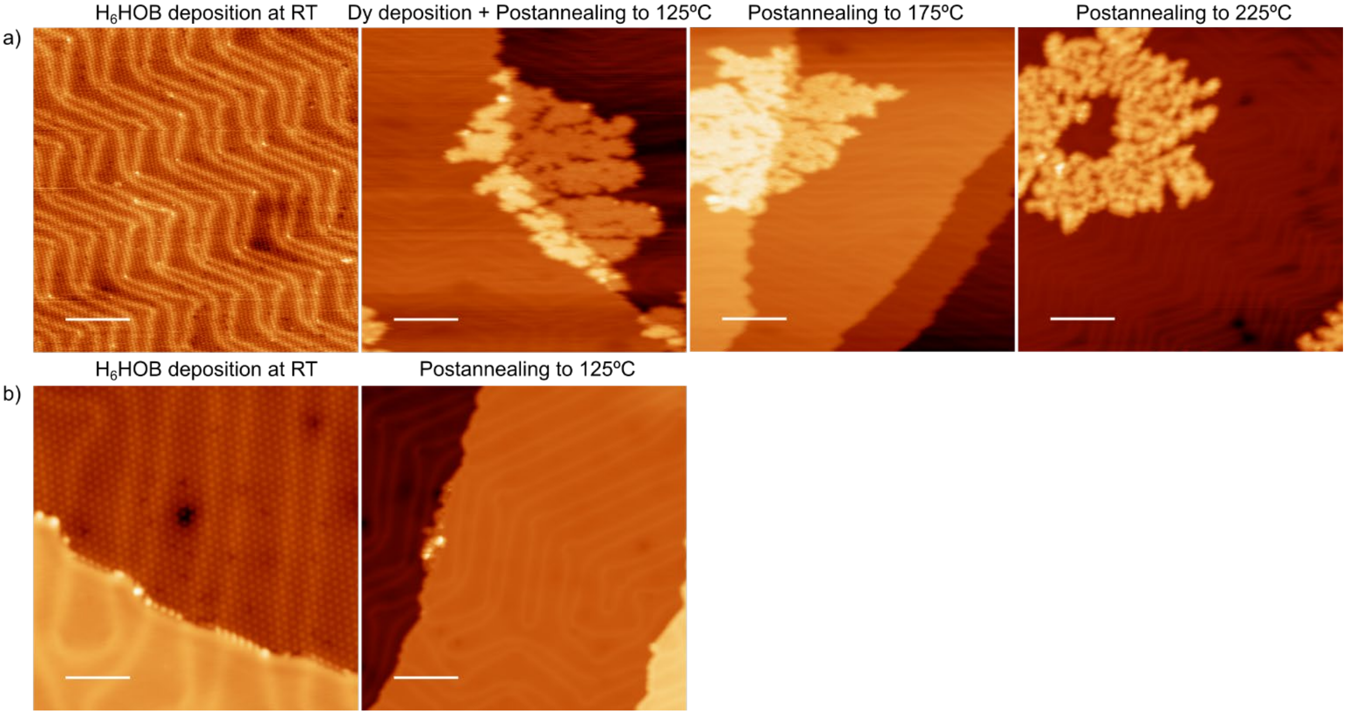

Figure S2: Tracking the desorption of H_6HOB and Dy(p-HOB)$_2$ from Au(111) upon annealing at increasing temperatures. (a) Tracking the desorption of hexahydroxybenzene when Dysprosium is added. Molecular islands still survive after post-annealing temperatures of 225ºC. Images were taken at 77 K. (b) Tracking the desorption of hexahydroxybenzene species without adding any lanthanide atom. After a post-annealing of 125ºC there is no fingerprint of the molecular self-assembly, indicating the full molecular desorption. The images have been taken at 4.4 K. Scale bars = 20 nm (a), 5 nm (left panel, b), and 10 nm (right panel, b). Scanning parameters: (a), from left to right, V_{bias} = 1.0 V, I_t = 100 pA, T = 77 K; V_{bias} = 0.2 V, I_t = 100 pA, T = 77 K; V_{bias} = 0.2 V, I_t = 100 pA, T = 77 K; V_{bias} = 0.5 V, I_t = 100 pA, T = 77 K; (b), from left to right: V_{bias} = 0.5 V, I_t = 100 pA, T = 4 K; V_{bias} = 0.5 V, I_t = 100 pA, T = 4 K.

III. Atomistic reaction pathways towards the on-surface formation of pristine Dy(H_6HOB)$_2$ and partially deprotonated Dy(p-HOB)$_2$ species.

The experimental evidence points out to the coexistence on the Au(111) surface of both pristine Dy(H_6HOB)$_2$ and partially deprotonated Dy(p-HOB)$_2$ species. On this basis, in order to propose plausible reaction mechanisms towards their on-surface formation and the preferential formation of one of the species over the other one, we have carried out a large battery of Density Functional Theory (DFT)-based calculations, in combination with the Gibbs free-energy formalism, to structurally and energetically propose viable formation mechanisms and characterize the different steps involved in each reaction path, as well as the transition states and associated energy barriers for each sub-reaction. Once the initial, intermediate and final structures have been established, we have adopted the Climbing-Image Nudged Elastic Band (CI-NEB) formalism [14], as implemented in the plane-wave simulation package QUANTUM ESPRESSO [15], to compute the minimum energy paths (MEPs) and transition state energy barriers for all the sub-reactions involved in the formation of the species (see Figure S3). These calculations have been performed for the periodic structures already described. Within the CI-NEB approach the initial, final, and a sufficient number of intermediate image-states (20 in the present case) for each sub-reaction were free to fully relax for the different systems.

On the basis of the results of these MEP and transition state energy barrier calculations, in conjunction with the Gibbs free energy of each structure, we can construct the full Gibbs free-energy profiles shown in Figure S3. For the on-surface formation of the Dy(p-HOB)$_2$ the calculated mechanism can progress involving two different routes. The first one proceeds by the capture of a Dy atom by the pristine H_6HOB molecule on the surface (a barrierless process with a net free-energy gain of -1.29 eV), followed by three sequential molecular surface-assisted deprotonations with barriers of 0.49, 0.54 and 0.58 eV, and net free-energy gains of -0.04, -0.06 and -0.06 eV, respectively. After that, the adsorbed Dy(p-HOB) (-3H) captures a partially deprotonated p-HOB (-3H) with a barrier of 0.19 eV, and a net free-energy gain of -2.64 eV completing the formation of the Dy(p-HOB)$_2$ (-6H). This mechanism yields a maximum barrier of 0.58 eV as a limiting reaction step (third surface-assisted deprotonation) and a high net free-energy gain of -4.09 eV, making the process highly probable in the experimentally applied temperature range.

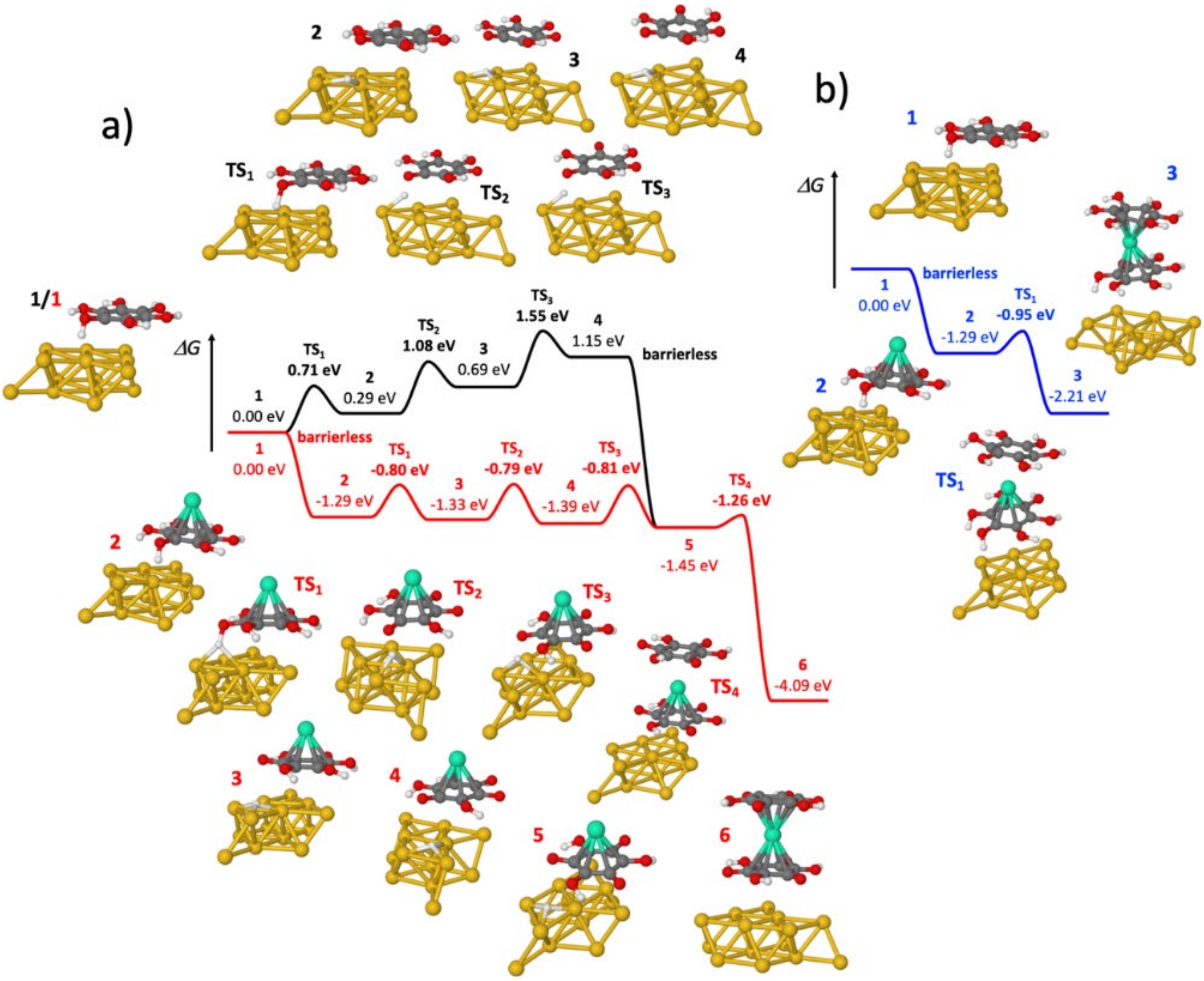

Figure S3. Mechanistic proposal for the on-surface formation of the: a) partially deprotonated Dy(p-HOB)$_2$, and b) pristine Dy(H_6HOB)$_2$ species. Optimized ground-state models corresponding to each reaction step, Gibbs free-energy profiles (in eV) by including entropic effects at 300 K, and transition states of the sub-reactions, for which non-null energy barriers have been found, are also shown.

The second route studied evolves by three direct surface-assisted molecular deprotonations with barriers of 0.71, 0.79 and 0.86 eV, and net free-energy losses of +0.29, +0.40 and +0.46 eV, respectively, towards the subsequent capture of a Dy atom, once triply deprotonated, in a barrierless process with a net free-energy gain of -2.60 eV. From this point the mechanism proceeds like in the previous case. For this second route, the free energy gain is again -4.09 eV, but the global energy barrier to be overpassed is 1.55 eV, which makes the process much more unlikely than for the previous route.

On the other hand, for the on-surface formation of the pristine $Dy(H_6HOB)_2$, the calculated mechanism progression involves: (i) the capture of a Dy atom by the pristine H_6HOB on the surface (the same barrierless process with a net free energy gain of -1.29 eV than for the first route of the previous case), followed by the capture of a pristine H_6HOB molecule, with a barrier of 0.34 eV and a net free-energy gain of -0.92 eV, to complete the on-surface formation of the pristine $Dy(H_6HOB)_2$. This mechanism yields a maximum barrier of 0.34 eV, but a net free-energy gain of -2.21 eV, a much lower value than that for the formation of the $Dy(p\text{-}HOB)_2$ in its most favorable scenario (-4.09 eV). This detailed theoretical analysis reinforces the possibility of finding both partially deprotonated $Dy(p\text{-}HOB)_2$ and pristine $Dy(H_6HOB)_2$ species coexisting on-surface, as evidenced by the experiments. Nonetheless, the predicted limiting step barriers in both processes are similar, while the net free-energy gain much favors the formation of the partially deprotonated species, with a higher probability to be found on the surface (see Figure S3).

IV. DFT results for $Dy(H_6HOB)_2$ and $Dy(p\text{-}HOB)_2$ species on Au(111).

Two scenarios were considered for the DFT calculations: (i) pristine molecules with all the -OH groups intact, $Dy(H_6HOB)_2$; (ii) 50% deprotonated molecules, i.e., each initial H_6HOB molecule preserves only three -OH groups, totalizing six intact -OH groups in the sandwich structure, $Dy(p\text{-}HOB)_2$. The DFT optimized models for the two scenarios are presented in Figure S4. In both cases the calculations were performed considering three $Dy(H_6HOB)_2$ or $Dy(p\text{-}HOB)_2$ molecules per unit cell, and the unit cells are represented by dashed lines in Figure S4.

In the case of scenario (i), the species are physisorbed, lying at 3.52 Å above the surface, with an adsorption energy of 0.23 eV per molecule and featuring an on-hollow preferential absorption. Although the molecules are lying almost flat, steric hindrance makes some -OH to rotate towards the surface, so the lowest ring of the molecules is slightly inclined respect to the Au(111) surface (see Figure S3a). In this situation, the molecules are very weakly interacting with the surface displaying an intermolecular interaction of 0.95 eV per molecule. In the scenario (ii), the molecules are physisorbed at 3.35 Å above the surface, with an adsorption energy of 0.12 eV per molecule and an on-hollow preferential absorption. In this situation the molecules are perfectly planar on the surface and there is a strong intermolecular interaction resulting in 2.10 eV per molecule (see Figure S4b). It is interesting to mention that structural relaxations of interfaces with molecules with different chirality result in instability independently on the molecular deprotonation degree considered.

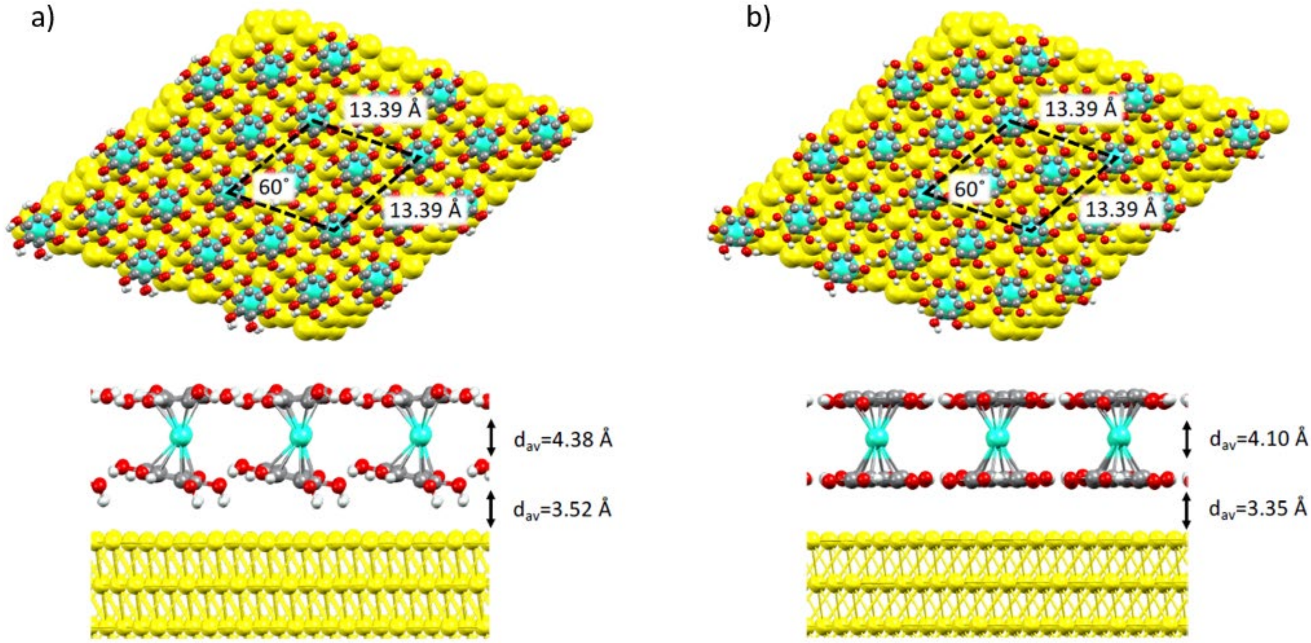

Figure S4. Top and side views of the optimized DFT models for (a) pristine $Dy(H_6HOB)_2$ molecules on Au(111); (b) 50% deprotonated $Dy(p\text{-}HOB)_2$ molecules on Au(111). Yellow, cyan, red, grey and white balls represent Au, Dy, O, C and H atoms, respectively.

Figure S5a, b presents simulated STM images for the two DFT models, which is compared with an experimental STM image (Figure S5c). The rotation of the -OH groups in the case of pristine molecules leads to an asymmetry in the simulated STM image of the molecules, that present an oblate shape (see Figure S5a). For the partially deprotonated molecules, the simulated STM image shows perfectly round molecules (see Figure S5b), in agreement with the experimental images, where round molecules where observed (see Figure S5c). The saturation of colour, brightness and contrast is the same in both simulated images, to allow a direct comparation. When comparing figures S5a and S5b, the molecules present different contrast, being the pristine species brighter at the selected bias voltage. In the experimental STM images it is possible to observe molecules with different degrees of brightness. Thus, the comparison between the DFT calculations and the experimental results indicate that the molecules observed in the experimental STM images can have distinct degrees of deprotonation.

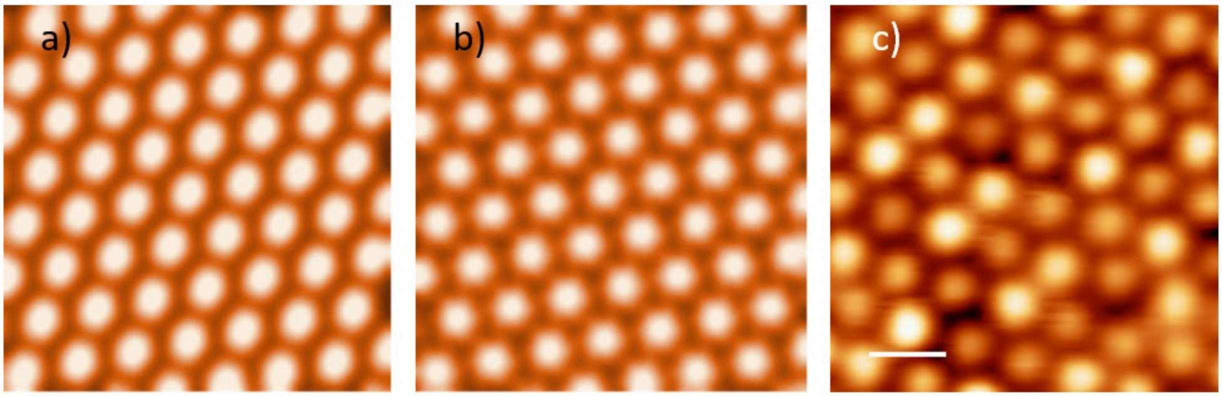

Figure S5. (a) DFT simulated STM image of pristine $Dy(H_6HOB)_2$ molecules. (b) DFT simulated STM image of 50% deprotonated $Dy(p\text{-}HOB)_2$ molecules. (c) Experimental STM image of a supramolecular assembly of $Dy(p\text{-}HOB)_2$ species on Au(111). Scale bar is 1.0 nm. Scanning parameters: V_{bias} = 500 mV, I_t = 100 pA, T = 4 K.

Additionally, to the calculations of the sandwich structure, we performed some test calculations of a structure consisting of a Dy atom underneath the H_6HOB molecule (See Figure S6). In this case the system is very weakly bonded, with a bonding energy per molecule of only 0.47 eV in the case of pristine molecules, while the $Dy(H_6HOB)_2$ has a bonding energy of 0.83 eV per molecule. For the partially deprotonated molecules, the structure is not stable for any initial configuration.

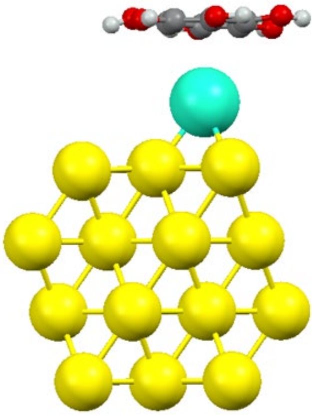

Figure S6. DFT simulation of a dysprosium atom intercalated under a H_6HOB molecule. Yellow, cyan, red, grey and white balls represent Au, Dy, O, C and H atoms, respectively.

V. Additional STS data and discussion

In order to have a more complete analysis, we acquired the STS data in three different manners: as a function of the lateral distance (Figure S7); only focusing at the brighter protrusions in the organometallic complexes (Figure S8) and only focusing on the darker protrusions in the islands (Figure S9). We perform these comparisons in either Dysprosium (Figure S7) or Erbium (Figures S8,9) sandwiches. As it can be seen, no general trend is observed for these analyses. The band gap values (determined within the threshold of the resonant peaks) range between 0.5 eV and 1.5 eV for Er(p-HOB)$_2$ and 1.0 eV and 1.7 eV for Dy(p-HOB)$_2$. The resonant peak for Er(p-HOB)$_2$ shifts from -0.3 up to -1.7 eV for negative biases and from 0.6 up to 1.6 eV for positive biases, at different spots. For Dy(p-HOB)$_2$ the peaks shift from -0.7 up to -1.5 eV for negative biases and from 0.9 up to 1.8 eV for positive biases, at different spots. They have been assigned as the HOMO and LUMO of the systems.

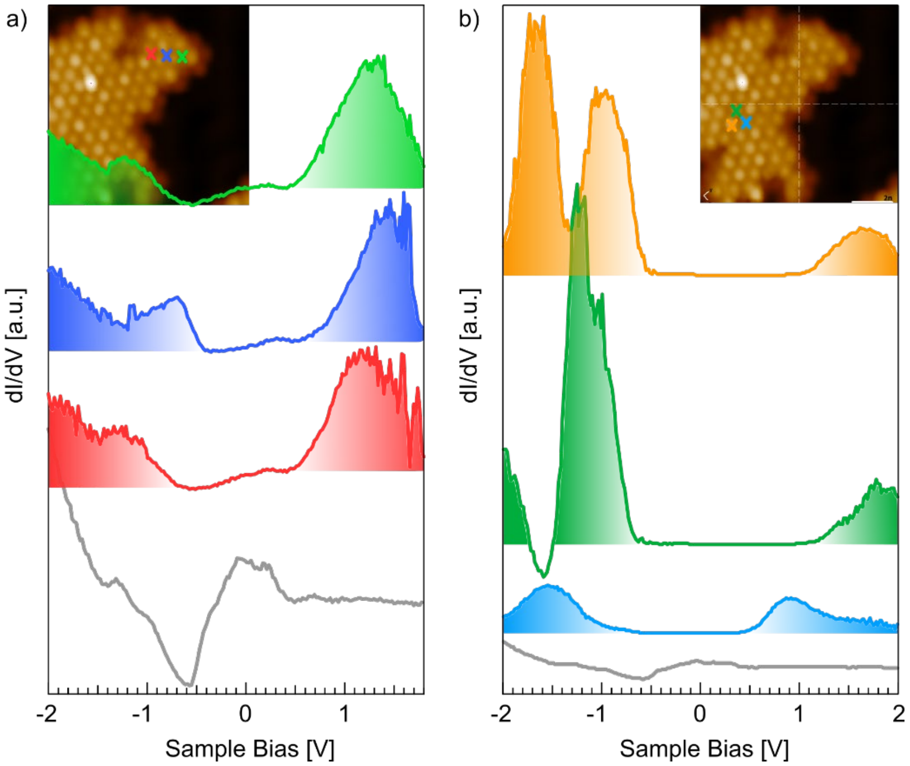

Figure S7: Point STS of a Dy(p-HOB)$_2$ island on Au(111). (a-b) dI/dV spectra acquired on selected positions. Scanning parameters of the STM image: V_{bias} = 0.1 V, I_t = 40 pA, T = 4 K.

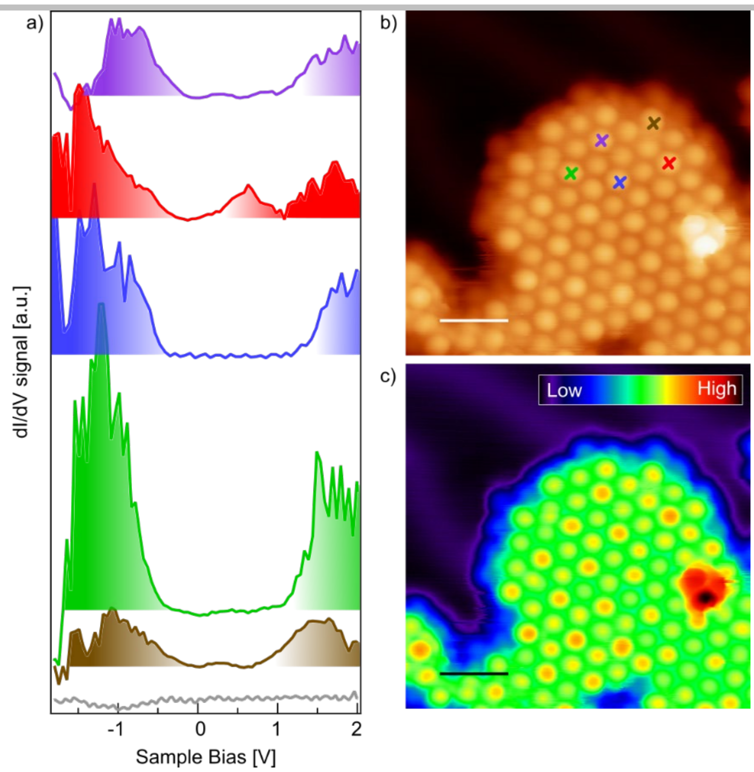

Figure S8: dI/dV spectra on the brighter protrusions of an Er(p-HOB)$_2$ island. (a) Point dI/dV spectra at each sandwich location. (b) STM image of the corresponding molecular island under study, indicating with colors crosses where the point dI/dV spectra were located; (c) Same STM image but using the 'Nih' WSxM color palette in order to highlight the brightest protrusions. dI/dV spectra has been interpolated (N=100). Scale bar = 2 nm. Scanning parameters of the STM image: V_{bias} = 0.5 V, I_t = 20 pA, T = 4 K.

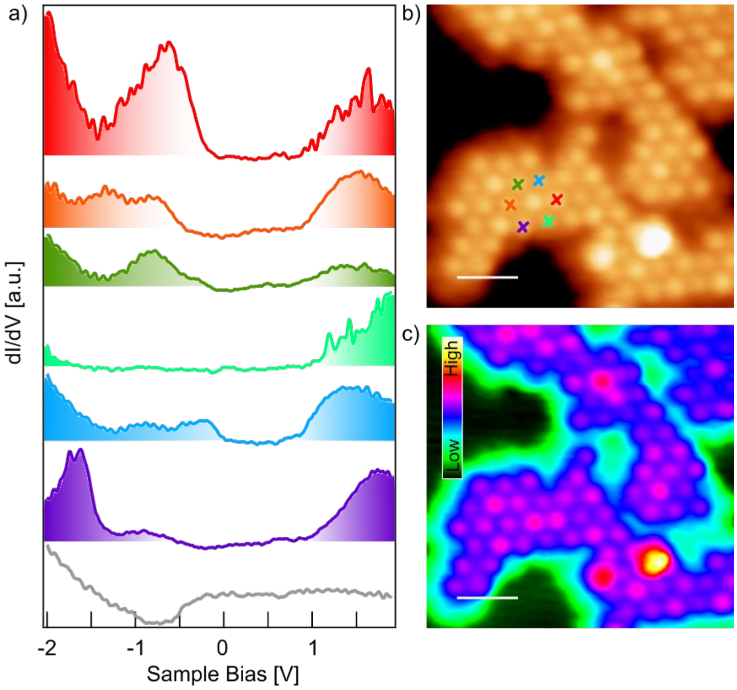

Figure S9: dI/dV spectra on the darker protrusions of an Er(p-HOB)$_2$ island. (a) Point dI/dV spectra at each sandwich location. (b) STM image of the corresponding molecular island under study, indicating with colors crosses where the point dI/dV spectra were located; (c) Same STM image but using the 'PseudoColor' WSxM colour palette in order to highlight the brightest from the darkest protrusions coming from the superlattice. dI/dV spectra has been smoothed with a binominal of order 3. Scale bars = 2 nm. Scanning parameters of the STM image: V_{bias} = 0.5 V, I_t = 10 pA, T = 4 K.

VI. Normalized magnetization curves

Figure S10 presents the magnetization curves of Dy(p-HOB)$_2$ normalized to the saturation intensity. It is possible to observe that at grazing incidence the magnetization curve saturates at a lower magnetic field than at NI.

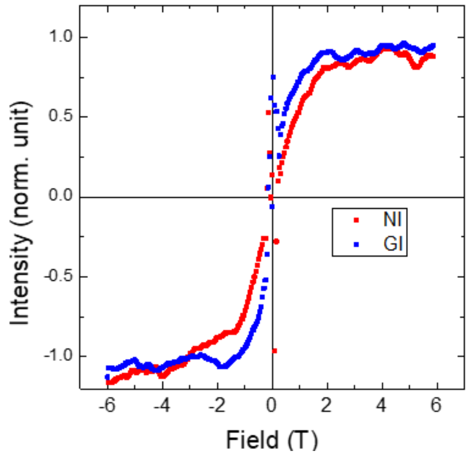

Figure S10. Magnetization curves of Dy(p-HOB)$_2$ normalized to have an intensity of 1.0 at saturation.

VII. Modelling of the charge orientation

Figure S11 presents models of the orientation of the charge on the lanthanide ions depending on the crystal field environment. Figures S11a, b represents Dy^{3+} and Er^{3+} ions on the crystal field of double-deckers where there is an eight-fold coordination. The Dy/Er atoms coordinate with four atoms from upper layer and four from the lower layer of the sandwich structure and there is a 45º relative rotation between the two layers. In this configuration the lanthanide ions are compressed which favours an out-of-plane alignment of the spins orientation for Dy^{3+} and in-plane for Er^{3+}, as indicated by the arrows.

Figures S11c, d represent the crystal field of the hexahydroxybenzene sandwiches where the Dy^{3+} and Er^{3+} ions are coordinated with six atoms from the upper layer and six from the lower layer of the sandwich. In this situation, the crystal field is more elongated due to the higher separation between the two molecular layers which favours an in-plane alignment of the spins for Dy^{3+} and out-of-plane for Er^{3+}.

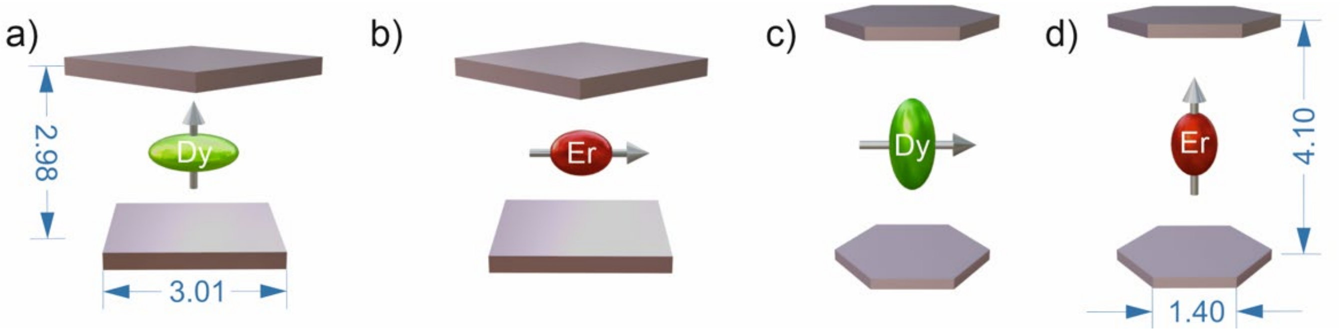

Figure S11. Schematic models of the placement of the lanthanide charge in the crystal field environment. (a) $DyPc_2$, (b) $ErPc_2$, (c) Dy(p-HOB)$_2$, (d) Er(p-HOB)$_2$. All distances are given in Å units.

References

[1] M. Schmid, H. Steinsrück, J. M. Gottfried, Surface and Interface Analysis **2014**, *46*, 505-511.

[2] a) B. T. Thole, P. Carra, F. Sette, G. van der Laan, *Phys. Rev. Lett.* **1992**, *68*, 1943-1946; b) P. Carra, B. T. Thole, M. Altarelli, X. Wang, *Phys. Rev. Lett.* **1993**, *70*, 694-697.

[3] Y. Teramura, A. Tanaka, B. T. Thole, T. Jo, *J. Phys. Soc. Jpn.* **1996**, *65*, 3056-3059.

[4] P. Giannozzi, S. Baroni, N. Bonini, M. Calandra, R. Car, C. Cavazzoni, D. Ceresoli, G. L. Chiarotti, M. Cococcioni, I. Dabo, A. Dal Corso, S. De Gironcoli, S. Fabris, G. Fratesi, R. Gebauer, U. Gerstmann, C. Gougoussis, A. Kokalj, M. Lazzeri, L. Martin-Samos, N. Marzari, F. Mauri, R. Mazzarello, S. Paolini, A. Pasquarello, L. Paulatto, C. Sbraccia, S. Scandolo, G. Sclauzero, A. P. Seitsonen, A. Smogunov, P. Umari, R. M. Wentzcovitch, *Journal of Physics: Condensed Matter* **2009**, *21*, 395502.

[5] J. P. Lewis, P. Jelínek, J. Ortega, A. A. Demkov, D. G. Trabada, B. Haycock, H. Wang, G. Adams, J. K. Tomfohr, E. Abad, H. Wang, D.A. Drabold, Phys. Status Solidi Basic Res. 2011, 248, 1989–2007.

[6] J. P. Perdew, A. Ruzsinszky, G. I. Csonka, O. A. Vydrov, G. E. Scuseria, L. A. Constantin, X. Zhou, K. Burke, *Phys Rev Lett* **2008**, *100*, 136406.

[7] M. De La Pierre, R. Orlando, L. Maschio, K. Doll, P. Ugliengo, R. Dovesi, *J Comput Chem* **2011**, *32*, 1775.

[8] G. Kresse, D. Joubert, *Phys Rev B* **1999**, *59*, 1758.

[9] S. Grimme, *J Comput Chem* **2006**, *27*, 1787.

[10] H. J. Monkhorst, J. D. Pack, *Phys Rev B* **1976**, *13*, 5188.

[11] F. J. Garcia-Vidal, J. Merino, R. Perez, R. Rincon, J. Ortega, *Phys. Rev. B* **1994**, *50*, 537–547.

[12] J. M. Blanco, F. Flores, R. Pérez, *Prog. Surf. Sci.* **2006**, *81*, 403-443.

[13] a) A.C. Gómez-Herrero, C. Sánchez-Sánchez, F. Chérioux, J.I. Martínez, J. Abad, L. Floreano, A. Verdini, A. Cossaro, E. Mazaleyrat, V. Guisset, P. David, S. Lisi, J.A. Martín-Gago, *Chem. Sci.* **2021**,*12*, 2257-2267; b) L. Giovanelli, O. Ourdjini, M. Abel, R. Pawlak, J. Fuj, L. Porte, J-M. Themin, S. Clair, *J. Phys. Chem. C*, **2014**, *118*, 14899-14904.

[14] a) *Classical and Quantum Dynamics in Condensed Phase Simulations*, (Eds. B. J. Berne, G. Ciccotti, D. F. Coker); World Scientific, **1998**; b) G. Henkelman, H. Jónsson, *J. Chem. Phys.* **2000**, *113*, 9978-9985; c) G. Henkelman, B. P. Uberuaga, H. Jónsson, *J. Chem. Phys.* **2000**, *113*, 9901-9904.

[15] P. Giannozzi, S. Baroni, N. Bonini, M. Calandra, R. Car, C. Cavazzoni, D. Ceresoli, G. L Chiarotti, M. Cococcioni, I. Dabo, *J. Phys.: Cond. Matter.* **2009**, *21*, 395502.

Author Contributions

S.O.P. and D.E. conceived and designed the experiments, supervised the project and led the collaboration efforts. S.K.M., S.O.P., M.T., L.C., D.M., B.M.C., C.N., M.A.V., P. G. and D.E. carried out the experiments and obtained the data. The experimental data were analyzed and discussed by all the authors. J.I.M. performed the DFT calculations. The manuscript was written by S.O.P., M.T. and D.E. with contributions from all the authors.
